# Generative diffusion models in infinite dimensions: a survey

**DOI:** 10.1098/rsta.2024.0322

**Published:** 2025-06-19

**Authors:** Giulio Franzese, Pietro Michiardi

**Affiliations:** ^1^ Data Science, EURECOM, Biot, France

**Keywords:** stochastic differential equations, generative diffusion models, Hilbert spaces

## Abstract

Diffusion models have recently emerged as a powerful class of generative models, achieving state-of-the-art performance in various domains such as image and audio synthesis. While most existing work focuses on finite-dimensional data, there is growing interest in extending diffusion models to infinite-dimensional function spaces. This survey provides a comprehensive overview of the theoretical foundations and practical applications of diffusion models in infinite dimensions. We review the necessary background on stochastic differential equations in Hilbert spaces, and then discuss different approaches to define generative models rooted in such formalism. Finally, we survey recent applications of infinite-dimensional diffusion models in areas such as generative modelling for function spaces, conditional generation of functional data and solving inverse problems. Throughout the survey, we highlight the connections between different approaches and discuss open problems and future research directions.

This article is part of the theme issue ‘Generative modelling meets Bayesian inference: a new paradigm for inverse problems’.

## 1. Introduction

Diffusion models have emerged as a prominent class of generative models, achieving notable success in various fields such as image generation [1], audio synthesis [2,3], video [4,5], molecular structures and general three-dimensional shapes [6–9] and conditional generation from open-ended text prompts [10–13]. For applications involving stochastic processes and complex systems where finite-dimensional approximations are insufficient, like weather forecasts or seismology [14,15] or extremely high-resolution point clouds [16], generative models which operate directly in function space are required. This survey aims to provide a structured overview of the theoretical and practical developments in generative diffusion models operating in infinite dimensions.

A fundamental motivation for considering infinite-dimensional approaches arises from Bayesian inverse problems in function spaces. The work in [17] established a rigorous mathematical framework for inference in such settings, emphasizing that naive finite-dimensional approximations often introduce undesirable pathologies. Rather than discretizing early in the modelling process, a more principled approach is to *avoid discretization until the last possible moment* [17]. Numerous studies have demonstrated that working directly in infinite-dimensional spaces leads to improved stability and accuracy in inference tasks [18,19].

The necessity of such models stems from the intrinsic limitations of finite-dimensional approximations, particularly their inconsistency across different resolutions. In empirical studies on generative diffusion models, it has been widely observed that models trained at one resolution often fail to generalize across different resolutions or scales unless carefully adapted [20–23]. This issue extends beyond neural architecture design (e.g. parameter adjustments) and is fundamentally linked to the role of *trace-class noise* in the perturbation process [24–31]. When the additive noise has a flat spectral density, changing the sampling frequency (i.e. the resolution) typically alters the effective signal-to-noise ratio, affecting consistency in generative models.

In contrast, infinite-dimensional methods allow for the construction of generative models that generalize effectively across different resolutions and are not constrained by regular sampling grids [24–33]. Additionally, infinite-dimensional generative models have been shown to provide enhanced parameter efficiency [26]. These models inherently accommodate function spaces in a way that ensures robustness and consistency in inference and generation tasks, making them particularly well-suited for applications requiring flexibility across multiple scales.

The problem of generative modelling in functional domains can be approached by adapting the techniques valid for the finite-dimensional case to the one where observed data points are considered as finite-resolution realizations of underlying functions [34–36]. These approaches allow for the incorporation of the notion of underlying manifold (whether Euclidean space or not) on which the data is collected, without the need to explicitly consider the machinery of infinite-dimensional stochastic calculus [37]. However, scaling these methods to arbitrary resolution is challenging. A different approach, which has been incorporated by some of the purely functional domain diffusion models, is to consider pseudo-invertible mappings from infinite-dimensional domains onto finite-dimensional ones and consider classical diffusion in those finite spaces [38,39]. Given the limitations of such approaches, a purely function space investigation of diffusion processes has been explored by the community, which we describe in the following.

### (a) An overview of existing methods and applications

Although the primary focus of this survey is the exploration of *infinite*-dimensional diffusion models, we first provide an overview of key generative models based on diffusion and related dynamics in finite dimensions, to set the stage for a discussion of their infinite-dimensional counterparts and applications.

On the left column of table 1, we summarize the major classes of finite-dimensional generative models. Among these, *discrete-time* diffusion models, commonly referred to as DDPM [5,40,41], learn to approximate the reversal of a forward Markov chain with a finite number of steps. This forward process perturbs the original data, typically through additive Gaussian noise, which smooths the data distribution. A related approach, annealed Langevin dynamics, replaces single-step transitions with multiple runs of Langevin dynamics, using the perturbed score function as the drift term to iteratively refine the noisy samples [42,43].

**Table 1.** Finite-dimensional generative models and infinite-dimensional counterparts.

finite-dimensional	infinite-dimensional	position in survey
DDPM [5,40,41]	[28]	§4a
annealed-LD [42,43]	[30]	§3a
score-SDE [1]	[27,31,44,45]	§2a
flow matching [46,47]	[29]	§3b
diffusion bridges and stochastic control [48,49]	[32,33,50]	§5a
Bayesian inverse problems [51,52]	[24,25]	§5b

As the number of steps tends to infinity, the Markov chain formulation converges to a continuous-time setting, leading to score-based SDEs [1], which model the forward and reverse evolution through stochastic differential equations. Another approach, known as *Flow Matching* [46,47], models the perturbation dynamics deterministically, inducing transport flows that map between the initial noisy distribution and the target data distribution.

Beyond generative modelling, diffusion models have been applied to stochastic control problems and Bayesian inverse problems. In finite dimensions, diffusion bridges provide a framework for modelling stochastic paths conditioned on endpoints, making them useful in molecular dynamics and controlled trajectory planning [48,49]. Similarly, diffusion-based priors have been leveraged in Bayesian inverse problems, where they help reconstruct unknown samples from noisy measurements [51,52]. By providing strong probabilistic priors, these models enhance inference accuracy and enable uncertainty quantification in complex systems.

The transition to infinite-dimensional settings introduces additional mathematical and computational challenges, which we explore in later sections. In particular, we discuss the infinite-dimensional counterpart of score-SDEs [27,31,44,45] in §2a, where they appear as specific cases of equation (2.4). Alternative formulations, including annealed Langevin dynamics [30] and flow matching [28], are examined in §3a,b, in connection with equations (3.1) and (3.4). Applications of diffusion bridges and Bayesian inverse problems in infinite dimensions are covered in §5a,b, particularly in equations (5.2)–(5.4).

### (b) Structure of the manuscript

In §2, we delve into the formal definition of diffusion models in infinite dimensions, focusing on Hilbert spaces. We introduce the necessary mathematical foundations, such as SDEs in Hilbert spaces, the properties of infinite-dimensional diffusion processes and key concepts like time-reversal of SDEs and the formulation of probability measures. Special attention is given to generative processes driven by these models, alongside theoretical considerations such as existence and uniqueness of solutions. In §3, we explore alternative methodologies for constructing generative models in infinite-dimensional settings, contrasting them with the diffusion-based approaches discussed earlier. We review methods like annealed Langevin dynamics and functional flow matching and connect their mathematical underpinnings to diffusion models, highlighting both their strengths and limitations. In §4, we outline the loss functions and training objectives associated with these parametric methods, particularly in terms of denoising and likelihood maximization. We also briefly overview neural architectures suitable for functional data and analyse their ability to handle resolution invariance. In §5, we provide an overview of various real-world applications of infinite-dimensional diffusion models. Other than purely generative use cases, we discuss the solution of Bayesian inverse problems, stochastic bridges and optimal control.

Finally, in §6, we summarize the key takeaways from this survey, highlighting the theoretical advancements and practical applications of diffusion models in infinite dimensions. We also outline open problems and future research directions, suggesting ways to further refine these models and explore their broader use in generative modelling and related fields.

## 2. Diffusion-based generative models in Hilbert spaces

We consider H to be a real, separable Hilbert space with inner product ⟨⋅,⋅⟩ , norm $|\;|\cdot {|\;|}_{H}$ . Let L(H) be the set of bounded linear operators on H , B(H) be its Borel σ -algebra, $B_{b}(H)$ be the set of bounded B(H) -measurable functions H→ℝ and P(H) be the set of probability measures on (H,B(H)) .

Our focus is to survey existing *generative models* which leverage the properties of diffusion processes in Hilbert spaces. Informally, given a finite collection of samples drawn from an underlying probability measure μ∈P(H) , we consider different, but strictly related techniques, to *transform* a sample drawn from an *easy* measure into a sample whose measure coincides with the desired μ . Consider the following H -valued SDE whose initial conditions are drawn from μ


(2.1) $$\left\{ \begin{array}{l} \text{d}X_{t}=(AX_{t}+f(X_{t},t))\text{d}t+\text{d}W_{t},\\ X_{0}∼\mu =\mu \in P(H), \end{array}\right.$$

where t∈[0,T] , $W_{t}$ is a R -Wiener process on H (of *trace* class) defined on the quadruplet $\left(\Omega ,F,(F_{t}{)}_{t\geq 0},\mathbb{Q}\right)$ . In our definitions, Ω,F are the sample space and canonical filtration, respectively: we consider Ω to be $C^{1}([0,T])$ , that is the space of all continuous mappings [0,T]→H , and $X_{t}(\omega )=\omega (t),\omega \in \Omega$ to be the *canonical* process. The domain of f is D(f)∈B([0,T]×H) , where f:D(f)⊂[0,T]×H→H is a measurable map. f is assumed to be Lipschitz continuous. The negative, symmetric operator A:D(A)⊂H→H is the infinitesimal generator of a $C_{0}$ -semigroup exp⁡(tA) in H (t≥0) , and μ is a probability measure in H . By definition, the measure associated with equation (2.1) is indicated with ℚ . The *law* induced at time τ∈[0,T] by the canonical process on the measure ℚ is indicated with $\rho_{\tau }\in P(H)$ , where $\rho_{\tau }(S)=\mathbb{Q}(\{\omega \in \Omega :X_{\tau }(\omega )\in S\})$ , and S is any element of B(H) . We define the law associated with the process defined in equation (2.1), when initial conditions are deterministic ( $X_{0}=z$ ) as $\rho_{\tau }^{z}$ . Notice that, by disintegration properties,


(2.2) $$\text{d}\rho_{\tau }(x)=\int \text{d}\rho_{\tau }^{z}(x)\text{d}\mu (z).$$

Given a countable orthonormal basis $\{e^{k}{\}}_{k\in \mathbb{N}}$ for the Hilbert space H , we can always construct an isomorphism with the space of square summable sequences $l_{2}(\mathbb{N})$ . Since R is a trace class operator, there exists a complete orthonormal system in H [37] that *diagonalizes* R , i.e. $Re^{k}=r^{k}e^{k}$ , with $r^{k}$ a positive scalar, where we leverage the fact that R is a symmetric operator being a covariance. The covariance operator can be used to define the Cameron Martin space $H_{R}\overset{\text{ def}}{=}R^{\frac{1}{2}}H$ associated with H , where the corresponding induced scalar product is $\left\langle \cdot ,\cdot \rangle_{H_{R}}=\langle R^{-\frac{1}{2}}\cdot ,R^{-\frac{1}{2}}\cdot \right\rangle$ .

Then, it is useful to notice that equation (2.1) can also be expressed as an (infinite) system of stochastic differential equations, in terms of $X_{t}^{k}=\left\langle X_{t},e^{k}\right\rangle$ *,* as:


(2.3) $$\text{d}X_{t}^{k}=a^{k}(X_{t})+f^{k}(X_{t},t)\text{d}t+\text{d}W_{t}^{k},\;k\in N,$$

where we introduced the projection $a^{k}(x)=\sum_{j=1}^{\infty }a^{k,j}x^{j}$ , with $a^{k,j}\overset{\text{ def}}{=}\langle Ae^{j},e^{k}\rangle$ and Lebesgue measure to obtain densities from measures. Moreover, $\text{d}W_{t}^{k}=\langle dW_{t},e^{k}\rangle$ with covariance given by $E_{\mathbb{Q}}[W_{t}^{k}W_{s}^{j}]=\delta (k-j)r^{k}\min(s,t)$ , δ in the Kronecker sense. Under condition 2.1, the SDE equation (2.1) admits a *strong* solution [37].

**Condition 2.1.** (i) $R^{\frac{1}{2}}H\subset D(A)$ , (ii) $AR^{\frac{1}{2}}$ *is a Hilbert–Schmidt operator*, and (iii) μ(D(A))=1 . *Notice that this condition is trivially satisfied whenever* A *is a bounded operator*.

### (a) Time reversal of SDEs

The idea of generative models based on SDEs in the form of equation (2.1) is to consider the *time-reversal* of the stochastic process $X_{t}$ , namely the process ${\hat{X}}_{t}\overset{\text{ def}}{=}X_{T-t}$ . Under certain conditions, the process ${\hat{X}}_{t}$ can be shown to be a solution of a new SDE. When this is the case, simulation of its dynamics allows obtaining a sample from the desired measure ( ${\hat{X}}_{T}=X_{0}∼\mu$ ).

More formally, we require that the time reversal of the canonical process, ${\hat{X}}_{t}=X_{T-t}$ , is again a diffusion process, with distribution given by the *path-reversed* measure $\hat{Q}(\omega )$ , along with the reversed filtration $\hat{F}$ . In the finite-dimensional case (which corresponds to the case where the Hilbert space has finite dimensionality), several results exist which prove (under appropriate technical conditions) that the time reversal of SDEs in the form of equation (2.1) (although finite dimensional) has again expression in terms of an SDE, with a new drift term composed as $-a^{k}(x)-f^{k}(x,t)+r^{k}\frac{\partial }{\partial x^{k}}\log\left(\frac{\text{d}\rho_{\tau }(x)}{\text{d}x})\right)$ , where $\frac{\text{d}\rho_{\tau }(x)}{\text{d}x}$ is the *density* of the measure w.r.t. the Lebesgue measure dx [53,54]. However, the time reversal of an infinite-dimensional process is more involved than for the finite-dimensional case. Indeed, in infinite-dimensional spaces, there is no equivalent of the Lebesgue measure to obtain densities from measures (i.e. dx in ${\mathbb{R}}^{\mathbb{N}}$ *does not exist* [55]). However, in the infinite-dimensional case, we can consider the single-dimensional conditional density $\frac{\text{d}\rho_{\tau }(x^{i}|x^{j\neq i})}{\text{d}x^{i}}$ (provided its existence), $\text{d}x^{i}$ being the single-dimensional Lebesgue measure. At a rather informal level, the reader can understand why considering such objects can alleviate the problem of non-existence of the desired densities in infinite dimensions: in the finite-dimensional case, thanks to disintegration of measures, the second term of the time-reversed drift can be simplified as $\frac{\partial }{\partial x^{k}}\log\left(\frac{\text{d}\rho_{\tau }(x^{k}\;|\;x^{j\neq k})}{\text{d}x^{k}}\frac{\text{d}\rho_{\tau }(x^{j\neq k})}{\text{d}x^{j\neq k}}\right)=\frac{\partial }{\partial x^{k}}\log\left(\frac{\text{d}\rho_{\tau }(x^{k}\;|\;x^{j\neq k})}{\text{d}x^{k}}\right)$ .

It is then possible to derive results for the infinite-dimensional case which are analogous to the finite-dimensional one. There are two major approaches to guarantee the existence of the reverse diffusion process. The first approach [56] relies on a finite local entropy condition. The second approach is based on stochastic calculus of variations [57], which we use to claim what follows.

**Theorem 2.1**. *Consider* equation (2.1). *Suppose that the conditions listed in Theorem 4.3 of* [57] *hold*. *Then* ${\hat{X}}_{t}$ *, corresponding to the path measure* $\hat{Q}(\omega )$ *, has the following SDE representation:*


(2.4) $$\left\{ \begin{array}{l} \text{d}{\hat{X}}_{t}=\left(-A{\hat{X}}_{t}-f({\hat{X}}_{t},T-t)+R𝛁\log\rho_{T-t}({\hat{X}}_{t})\right)\text{d}t+\text{d}{\hat{W}}_{t},\\ {\hat{X}}_{0}∼\rho_{T}, \end{array}\right.$$

*where* $\hat{W}$ *is a* $\hat{Q}$ R -*Wiener process and the notation* $R𝛁\log\rho_{t}(x)$ *stands for the mapping* H→H *that, when projected, satisfies* $\langle R▽\log\rho_{t}(x),e^{k}\rangle =r^{k}\frac{\partial }{\partial x^{k}}\log\left(\frac{\text{d}\rho_{t}(x^{k}|x^{j\neq k})}{\text{d}x^{k}}\right)$ . By projecting onto the eigenbasis, we have an infinite system of SDEs:


(2.5) $$\text{d}{\hat{X}}_{t}^{k}=\left(-a^{k}({\hat{X}}_{t})-f^{k}({\hat{X}}_{t},T-t)+r^{k}\frac{\partial }{\partial x^{k}}\log\left(\frac{\text{d}\rho_{T-t}({\hat{X}}^{k}|{\hat{X}}^{j\neq k})}{\text{d}x^{k}}\right)\right)\text{d}t+\text{d}{\hat{W}}_{t}^{k},\;k\in N.$$

Notice that strictly speaking, using the symbol ▽ is an abuse of notation, since we cannot identify it with the gradient (in Hilbert space) of a single function.

The result in [57] is remarkably general and elegant, but this generality comes at the cost of increased complexity in verifying that the necessary technical conditions hold. Although not the most general case, a concrete example of conditions that satisfy the coefficient assumptions outlined in [57] are those considered in [26], which we report hereafter, as discussed in detail in appendix A:

**Condition 2.2.** f=0 *and* A *is diagonal in* $\left\{e^{i}\right\}$ *, i.e.* $a^{i,j}=\delta_{i,j}a^{i}$ .

**Condition 2.3.** *Condition 2.2 is satisfied, and* A *is bounded:* $-a^{i}\leq M$ *, with* M *a finite positive constant*.

It is worth mentioning that much effort has been put in by the authors in [31] to generalize the settings considered here for the dynamics in equation (2.1), in particular with the possibility of considering non-constant operators $G_{t}$ , which precondition the Wiener process via $G_{t}\text{d}W_{t}$ . In their work, they focus on the time reversal formula at the level of the generator directly: in general, the existence of time reversal of a process and the validity of time reversal formulas do not imply each other. Such an approach is useful for investigating the relationship between different methodologies, as we will explore later.

For the validity of the methodologies described above, it is required that the conditional densities exist. Next, we introduce a different condition, which will be used to prove the existence of such densities and is at the root of the different generative modelling techniques explored in this survey, the *trait d’union* between all the technical requirements of infinite-dimensional diffusion models.

**Condition 2.4.** *For each* t∈(0,T] , *the measure* $\rho_{t}$ *is absolutely continuous with respect to the measure* $\rho_{t}^{0}$ .

In our introduction, we considered the generic nonlinear ( f≠0 ) SDE equation (2.1). However, in almost all actual applications in the literature, the simple linear case where f=0 is considered. This assumption allows a simpler investigation of the validity of time reversal equations (see appendix A). Furthermore, this has tremendous practical benefits, as under such conditions, there exists a weak solution to equation (2.1) as


(2.6) $$\begin{array}{lcr} & X_{t}=\exp(tA)X_{0}+\int_{0}^{t}\exp((t-s)A)\text{d}W_{s}, & \end{array}$$

which is typically amenable to cheap simulation. In this case, the conditional distribution admits a simple expression $\text{d}\rho_{t}^{z}(x)=N_{\exp(tA)z,S(t)}(x)$ , where $S(t)=\left(\int_{0}^{t}\exp(sA)R\exp(sA)\text{d}s\right)$ .

Condition 2.2 is particularly helpful in practical implementations, simplifying the computation of terms in the form AR , which would generally require working with a different basis and consequently induce extremely high computational cost. For simplicity of exposition in relating different works, we will often consider this assumption in our writing.

Under condition 2.2, $\langle S(t)e^{j},e^{i}\rangle \overset{\text{ def}}{=}s^{ij}(t)=\frac{\exp(2a^{i}t)-1}{2a^{i}}r_{i}\delta_{ij}$ . This simplified expression is central in the works [24,27,45] where reverse time dynamics are proved directly with approaches that do not explicitly require the existence of the conditional densities. Furthermore, in this particular case, the measures $\text{d}\mu_{t}^{0}(x)$ have a simple Gaussian expression $\text{d}N_{0,S(t)}(x)$ . In particular, in the works [24,25,27], it is assumed that $Ax=-\frac{x}{2}$ , and consequently, $RS^{-1}(t)=1-\exp(-t)$ , $R▽\log\rho_{T-t}(x)=-(1-\exp(-t){)}^{-1}\left(x-\exp\left(-\frac{t}{2}\right)E_{Q}\left[X_{0}\;|\;X_{t}=x\right]\right)$ . The proof strategy in these works revolves around the conditional expectation directly, without the need to explicitly define conditional densities. This is an advantage over the conditions imposed, e.g. in [56,57], which are designed for generally nonlinear processes. In particular, the key idea adopted for the proofs is to define projected, low-dimensional dynamics for the process $X_{t}$ , via $P^{D}(X_{t})$ , where $P^{D}$ is the projection operator on the first D basis vectors of H . Then, the existence of a backward process with a finite-dimensional expected value is proven using classical results. The core technical contribution is to show that as D→∞ the processes remain well-behaved and converge to the original infinite-dimensional dynamics, allowing us to claim that equations in the form of equation (2.4) (with the substitution of equation (2.7)) are valid generative models. Summarizing, the linear case has the following important property

**Theorem 2.2.** *Assume that* $\mu (R^{\frac{1}{2}}H)=1$ *and condition* 2.2 *holds. Then*

(i) *Condition 2.4 holds (* $\frac{\text{d}\rho_{t}}{\text{d}\rho_{t}^{0}}$ *exists).*
(ii) *The conditional densities* $\frac{\text{d}\rho_{t}(x^{i}\;|\;x^{j\neq i})}{\text{d}x^{i}}$ *exist.*
(iii)
(2.7) $$R▽\log\rho_{t}(x)=-RS(t{)}^{-1}\left(x-\exp(tA)E_{Q}\left[X_{0}\;|\;X_{t}=x\right]\right),$$
(iv)
(2.8) $$R▽\log\left(\frac{\text{d}\rho_{t}}{\text{d}\rho_{t}^{0}}\right)(x)=\exp(tA)E_{Q}[X_{0}\;|\;X_{t}=x].$$

*Proof*.

(i) Consider temporarily the assumptions $\mu (R^{\frac{1}{2}}H)=1$ and $\exp(tA)R^{\frac{1}{2}}H\subseteq S^{\frac{1}{2}}(t)H$ . Condition 2.4 is equal to $\rho_{t}^{0}(B)=0\Rightarrow \rho_{t}(B)=0$ for any measurable set B and any t∈(0,T] . Since $\rho_{t}(B)=\int \rho_{t}^{z}(B)\text{d}\mu (z)=0$ , the implication is true if $\rho_{t}^{z}\ll \rho_{t}^{0}$ for μ a.e. z . Given that both measures are Gaussian with means exp⁡(tA)z and 0 , respectively, and covariance S(t) the absolute continuity holds if $\exp(tA)z\in S^{\frac{1}{2}}(t)H$ for μ a.e. z . Given that $\mu (R^{\frac{1}{2}}H)=1$ , this is equivalent to $\exp(tA)R^{\frac{1}{2}}y\in S^{\frac{1}{2}}(t)H$ for all y∈H , which is exactly the assumption $\exp(tA)R^{\frac{1}{2}}H\subseteq S(t)H$ . We now prove that $\exp(tA)R^{\frac{1}{2}}H\subseteq S^{\frac{1}{2}}(t)H$ holds true if condition 2.2 holds. First, let us notice that the assumption is equivalent to assuming that $\forall y\in H,\exists z\in H:\exp(tA)R^{\frac{1}{2}}y=S^{\frac{1}{2}}(t)z$ . In this case, $S_{t}^{\frac{1}{2}}=((1-\exp(2tA))(-2A{)}^{-1}R{)}^{1/2}$ . Given a single y∈H , clearly $z=(-2A{)}^{\frac{1}{2}}(1-\exp(2tA){)}^{-\frac{1}{2}}\exp(tA)y$ satisfies the equality. Furthermore, its norm is equal to $\left\|z{\|}^{2}=\langle y,-2A(1-\exp(2tA){)}^{-1}\exp(2tA)y\right\rangle$ $\;=\langle y,2A(\exp(-2tA)-1{)}^{-1}y\rangle$ $\;=\sum_{i}\frac{2a^{i}}{\exp(-2ta^{i})-1}(y^{i}{)}^{2}\leq \sum_{i}(y^{i}{)}^{2}<\infty$ .
(ii) Condition 2.4 implies that the Radon–Nikodym derivative exists and is well defined. By restriction, $\frac{\text{d}\rho_{t}(x^{j\neq i})}{\text{d}\rho_{t}^{0}(x^{j\neq i})}$ exists and is uniquely defined as well. Then, by disintegration $\frac{\text{d}\rho_{t}(x)}{\text{d}\rho_{t}^{0}(x)}=\frac{\text{d}\rho_{t}(x^{i}\;|\;x^{j\neq i})}{\text{d}\rho_{t}^{0}(x^{i}\;|\;x^{j\neq i})}\frac{\text{d}\rho_{t}(x^{j\neq i})}{\text{d}\rho_{t}^{0}(x^{j\neq i})}$ , from which we conclude the existence of $\frac{\text{d}\rho_{t}(x^{i}\;|\;x^{j\neq i})}{\text{d}\rho_{t}^{0}(x^{i}\;|\;x^{j\neq i})}$ . Based on this, $\rho_{t}^{0}(x^{i}\;|\;x^{j\neq i})=N_{0,s^{i}}(x^{i})$ . Since $\text{d}\rho_{t}(x^{i}\;|\;x^{j\neq i})\ll \text{d}N_{0,s^{i}}(x^{i})\ll \text{d}x^{i}$ , the existence of the densities is proven.
(iii) $\frac{\partial }{\partial x^{i}}\log\frac{\text{d}\rho_{t}(x^{i}\;|\;x^{j\neq i})}{\text{d}x^{i}}=(\frac{\text{d}\rho_{t}(x^{i}\;|\;x^{j\neq i})}{\text{d}x^{i}}{)}^{-1}\frac{\partial }{\partial x^{i}}(\frac{\text{d}\rho_{t}(x^{i}\;|\;x^{j\neq i})}{\text{d}x^{i}})$ . Furthermore, $\text{d}\rho_{t}(x^{i}\;|\;x^{j\neq i})=\int \text{d}\rho_{t}(x^{i}\;|\;x_{0},x^{j\neq i})$ $\text{d}\mu (x_{0}\;|\;x^{j\neq i})$ , where $\text{d}\rho_{t}(x^{i}\;|\;x_{0},x^{j\neq i})=\text{d}N_{\exp(ta_{i})x_{0}^{i},s^{i}(t)}(x^{i})$ which has density w.t.t $\text{d}x^{i}$ . Then, $\frac{\text{d}\rho_{t}(x^{i}\;|\;x^{j\neq i})}{\text{d}x^{i}}=\int N_{\exp(ta_{i})x_{0}^{i},s^{i}(t)}(x^{i})\text{d}\mu (x_{0}^{i}\;|\;x^{j\neq i})$ , and $\frac{\partial }{\partial x^{i}}(\frac{\text{d}\rho_{t}(x^{i}\;|\;x^{j\neq i})}{\text{d}x^{i}})=-\int \frac{x^{i}-\exp(ta_{i})x_{0}^{i}}{s^{i}(t)}N_{\exp(ta_{i})x_{0}^{i},s^{i}(t)}(x^{i})$ $\text{d}\mu (x_{0}^{i}\;|\;x^{j\neq i})$ . Upon simple algebraic manipulation, we can get the desired result (the proof is based on the content of [24,26,33]).
(iv) Due to the Feldman–Hajek theorem [37]

$$\frac{d\rho_{t}^{z}}{\;d\rho_{t}^{0}}(x)=\exp\left(\left\langle R^{-1}x,\exp(tA)z\right\rangle -1/2{\left\|R^{-1/2}\exp(tA)z\right\|}^{2}\right).$$

Then, $R▽\frac{\text{d}\rho_{t}^{z}}{\text{d}\rho_{t}^{0}}(x)=\frac{\text{d}\rho_{t}^{z}}{\text{d}\rho_{t}^{0}}(x)\exp(tA)z$ . Consequently, $R▽\frac{\text{d}\rho_{t}}{\text{d}\rho_{t}^{0}}(x)=R▽\int \frac{\text{d}\rho_{t}^{z}}{\text{d}\rho_{t}^{0}}(x)\text{d}\mu (z)=$ $\int \frac{\text{d}\rho_{t}^{z}}{\text{d}\rho_{t}^{0}}(x)\exp(tA)z\text{d}\mu (z)=\int \exp(tA)z\text{d}\mu (z\;|\;X_{t}=x)\frac{\text{d}\rho_{t}}{\text{d}\rho_{t}^{0}}(x)$ and equation (2.8) holds (see [30] for further insights).

∎

If the linear operator A satisfies determinate spectral conditions, then $\rho_{t}^{z}∼\rho_{\infty }$ , for all z∈H and t>0 , where both measures have known Gaussian form [37,58–60]. Consequently, $\rho_{t}^{z}∼\rho_{\infty }∼\rho_{t}^{0}$ for all z∈H , which implies $\rho_{t}^{0}(B)=0\to \rho_{t}(B)=\int \rho_{t}^{z}(B)\text{d}\mu (z)=0$ , i.e. §2.4 holds ( $\frac{\text{d}\rho_{t}}{\text{d}\rho_{t}^{0}}$ exists). Unfortunately, this result, which is stronger than condition §2.4, does not hold for the most commonly used operators A , like −I , as it is required $\exp(tA)z\in S_{t}^{\frac{1}{2}}H$ , for z∈H generic ([60]).

Theorem 2.1 stipulates that, given some conditions, the reverse time dynamics of equation (2.1) exist in the form of SDE. However, for generative modelling purposes, the content of theorem 2.1 is stronger than necessary. What suffices is that there exists a new SDE whose time-varying law corresponds to the backward in time of the original $\rho_{t}$ . Clearly, if a time reversal exists, such a process satisfies the weaker measure matching condition.

To expand on this point and connect together the different generative modelling techniques discussed in the literature, it is necessary to introduce the Kolmogorov operators [31,61–64]


(2.9) $$L_{0}u(x,t)=D_{t}u(x,t)+\underset{Lu(x,t)}{\underbrace{\frac{1}{2}\text{Tr}RD_{x}^{2}u(x,t)+\langle Ax+f(x,t),D_{x}u(x,t)\rangle }},\;x\in H,t\in [0,T],$$

where $D_{t}$ is the time derivative, $D_{x},D_{x}^{2}$ are first- and second-order Fréchet derivatives in space (since both the scalar product and the trace are defined on the Hilbert space H , and not its dual, the quantities in the equation above should be interpreted after Riesz mapping M∘ ). The domain of the operator is assumed to be the set of smooth cylinder functions of finite support [31,61].

Provided appropriate conditions are satisfied, see for example [61,62], the time-varying measure $\text{d}\rho_{t}(x)$ exists, is unique, and solves the Fokker–Planck equation


(2.10) $$\begin{array}{lcr} & \int_{H}u(x,0)\text{d}\mu (x)+\int_{0}^{T}\text{d}s\int_{H}L_{0}u(x,t)\text{d}\rho_{s}(x)=0. & \end{array}$$

The result of theorem 2.1 can be thus understood at the level of time reversal of measures: by simply considering the negative of the operator L , we obtain $\frac{1}{2}\text{Tr}\{-RD_{x}^{2}u(x)\}+\langle -Ax,D_{x}u(x)\rangle$ . While −A can be associated with a new drift term, it is not possible to construct a Brownian motion term with covariance −R . However, $-\frac{1}{2}\text{Tr}\{RD_{x}^{2}u(x)\}=-\text{Tr}\{RD_{x}^{2}u(x)\}+\frac{1}{2}\text{Tr}\{RD_{x}^{2}u(x)\}$ . As shown in [31, App. B.2],


(2.11) $$-\int_{H}\text{Tr}\{RD_{x}^{2}u(x,t)\}\text{d}\rho_{s}(x)=\int_{H}\langle R▽\log\rho_{s}(x),D_{x}u(x,s)\rangle \text{d}\rho_{s}(x),$$

which allows us, as anticipated, to prove in a simpler way a *weaker* form of the content of theorem 2.1: an SDE in the form of equation (2.4), defined on a proper probability space, induces a time-varying measure which corresponds to the time-reversal of the original $\rho_{t}$ , i.e. the measure associated with equation (2.1). Furthermore, the equality in equation (2.11) allows us to claim that the measure $\rho_{t}$ is also a solution of the continuity equation (see [63–65] for definiteness and uniqueness)


(2.12) $$\int_{H}u(x,0)\text{d}\mu (x)+\int_{0}^{T}\text{d}s\int_{H}\left(\frac{\text{d}u(x,t)}{\text{d}t}+\langle Ax+f(x,t)-\frac{R}{2}▽\log\rho_{t}(x),D_{x}u(x,t)\rangle \right)\text{d}\rho_{t}(x)=0,$$

which corresponds to an ordinary differential equation (ODE) in the Hilbert space with deterministic drift


(2.13) $$\left\{ \begin{array}{l} \text{d}X_{t}=AX_{t}+f(X_{t},t)-\frac{R}{2}▽\log\rho_{t}(X_{t})\text{d}t\\ X_{0}∼\mu \end{array}\right..$$

These facts allow us to build a connection between the methodology discussed so far and other prominent approaches from the literature, as shown hereafter.

## 3. Alternative generative approaches

In §2, we introduced a family of generative models rooted in (variants) of the time reversal formula, shown in equation (2.4). As anticipated, inverting the dynamics of a diffusion process is a sufficient, but not necessary, strategy to obtain a generative model in function space. In this section, we briefly overview some different methodologies which appeared in the literature, clearly connecting their working mechanism and underlying assumptions with the ones described previously.

### (a) Annealed Langevin dynamics

Consider the N+1 dimensional sequence of measures $\rho_{0},\rho_{T/N},\ldots \rho_{T}$ . Suppose we have access to computational schemes which allow us, starting from a sample drawn from $\rho_{(i+1)T/N}$ , to obtain a sample from the target $\rho_{iT/N}$ . Then, a valid generative modelling technique is to run sequentially such computational schemes on the reverse order sequence. One particular approach [30] relies on running multiple annealed chains of Langevin sampling schemes, in the following fashion:


(3.1) $$\text{d}{\tilde{X}}_{t}=-{\tilde{X}}_{t}\text{d}t+S(iT/N)▽\log\left(\frac{\text{d}\rho_{iT/N}}{\text{d}\rho_{iT/N}^{0}}({\tilde{X}}_{t})\right)+\sqrt{2S(iT/N)R^{-1}}\text{d}{\tilde{W}}_{t}$$

where ${\tilde{W}}_{t}$ has covariance R . Dynamics in the form of equation (3.1) have the following fundamental property: their time-invariant measure is $\rho_{iT/N}$ ρiT/N [19,66]. Notice that for the scheme to be valid, the Radon–Nikodym derivative should be valid, i.e. condition 2.4 should hold, as required explicitly by the authors of [30]. Consequently, by selecting the sequence of measures as the law of a diffusion process in the form of equation (2.1), with the conditions described in theorem 2.2, the condition is satisfied, allowing comparison of the methods on the same ground.

To connect this scheme with the one described in [30], it is necessary to notice that $M\circ D_{x}^{\left(H_{S(iT/N)}\right)}\log\left(\frac{\text{d}\rho_{iT/N}}{\text{d}\rho_{iT/N}^{0}}\right)={▽}^{\left(H_{S(iT/N)}\right)}\log\left(\frac{\text{d}\rho_{iT/N}}{\text{d}\rho_{iT/N}^{0}}\right)=S(iT/N){▽}^{H}\log\left(\frac{\text{d}\rho_{iT/N}}{\text{d}\rho_{iT/N}^{0}}\right)$ . The iterative procedure that starts from a sample $∼\rho_{(i+1)T/N}$ , simulates dynamics equation (3.1) for a sufficiently long time (to reach approximately steady state) and obtains a sample from $\rho_{iT/N}$ , is consequently a valid generative model for μ .

Our goal hereafter is to discuss more clearly the connections between this approach and the schemes described previously. Considering a simple time-rescaling argument, it can be shown [37] that the dynamics in equation (3.1) are equivalent to


(3.2) $$\text{d}{\tilde{X}}_{t}=-\frac{1}{2}S(iT/N{)}^{-1}R{\tilde{X}}_{t}\text{d}t+\frac{R}{2}▽\log\left(\frac{\text{d}\rho_{iT/N}}{\text{d}\rho_{iT/N}^{0}}({\tilde{X}}_{t})\right)\text{d}t+\text{d}{\tilde{W}}_{t},$$

where ${\hat{W}}_{t}$ is a Brownian motion with covariance R . Then, under the assumptions of theorem 2.2, equation (3.2) reads


(3.3) $$\text{d}{\tilde{X}}_{t}=\frac{R}{2}▽\log(\rho_{iT/N}({\hat{X}}_{t}))\text{d}t+\text{d}{\hat{W}}_{t},$$

which is reminiscent of a classical Langevin equation in ${\mathbb{R}}^{N}$ . Clearly, equation (3.3) preserves the measure $\rho_{iT/N}$ . The reverse time dynamics described by equation (2.5) can then be interpreted as the schemes which anneal through the infinite ( N→∞,[1,T/N,…,T]→[0,T] ) sequences as follows:


$$d{\hat{X}}_{t}=\underset{\text{Equation (2.13). Transports }\rho^{T-t}\text{ into }\rho^{T-dt-t}}{\underbrace{\left(A{\hat{X}}_{t}+\frac{R}{2}\nabla \log\left(\rho_{T-t}\left({\hat{X}}_{t}\right)\right)dt\right)}}+\underset{\text{Equation (3.3) with }i/N=T-t\text{. Preserves }\rho^{T-t}}{\underbrace{\left(\frac{R}{2}\nabla \log\left(\rho_{T-t}\left({\hat{X}}_{t}\right)\right)dt+d{\hat{W}}_{t}\right)}}.$$

Furthermore, the approach in [30] can also be directly linked with the denoising approach of [24,25,27] thanks to the result in equation(2.8), which allows us to appreciate the connection among all the methods and a *denoising* interpretation of the generative dynamics.

### (b) Flow matching

It is worth mentioning that, in the literature, a purely deterministic approach to the problem (a generalization of the ODE equation (2.13)), built as a generalization of the finite-dimensional *Flow Matching* approach of [46], has been explored in [29]. In this work, the authors avoid the issues associated with time reversal by directly constructing a path of probability measures $({\hat{\rho }}_{t}{)}_{t\in [0,T]}$ which *anneals* from a tractable initial measure ${\hat{\rho }}_{0}$ (a Gaussian measure for example) to the target data distribution ${\hat{\rho }}_{T}=\mu$ . This path is constructed by flowing the initial measure along a time-dependent vector field v(x,t) on the Hilbert space H :


(3.4) $$\text{d}{\tilde{X}}_{t}=v(X_{t},t)\text{d}t,\;{\tilde{X}}_{0}∼{\hat{\rho }}_{0}=N_{0,C},$$

with C being a given trace class covariance. Since it represents a deterministic evolution, equation (3.4) can equivalently be described by a flow mapping ${\tilde{X}}_{t}=\phi ({\tilde{X}}_{0},t)$ *,* which is usually the space in which the design of such models is performed. The evolution of ${\hat{\rho }}_{t}$ along v(x,t) is governed by the continuity equation (again for u in the set of cylinder test functions)


(3.5) $$\int_{0}^{T}dt\int_{H}\left(\partial_{t}u(x,t)+\langle v(x,t)(x),{▽}_{x}u(x,t)\rangle \right)d{\hat{\rho }}_{t}(x)=0.$$

Notice that the difference between this methodology and the ones described previously is that the transformation of a Gaussian reference measure into the data measure takes place exactly in *finite time*, whereas for the other methodologies, this does not happen. Indeed, in equation (2.4), the initial conditions are drawn from $\rho_{T}$ , which can be close but not equal to a purely Gaussian distribution (unless μ is the steady-state distribution itself of the process, provided it exists, or T=∞ ). Similarly, the annealed Langevin approach requires running the last step of the chains for an infinite amount of time.

Given a conditional vector field v(x,t) *,* it is possible to compute the induced sequence of measures ${\hat{\rho }}_{t}$ . The inverse procedure, which is more important from a practical perspective (the annealed sequence is a design parameter and the field is unknown), is much more challenging. *A priori*, it is not even known whether, given a sequence of measures, a vector field which matches this sequence exists. In [29], the strategy for the construction of a sequence for which the field exists is split into steps: (i) selection of a family of vector fields $v_{t}^{z}$ which induce a flow of measures ${\hat{\rho }}_{t}^{z}$ whose starting point is ${\hat{\rho }}_{0}^{z}={\hat{\rho }}_{0}$ (thus independent from z ) and ending one (at time T ) is a measure *concentrated* around z , (ii) assumption that ${\hat{\rho }}_{t}^{z}\ll {\hat{\rho }}_{t}$ for μ a.e. z and almost every t∈[0,T] , and (iii) construction of $v(x,t)=\int v_{t}^{z}\frac{\text{d}{\hat{\rho }}_{t}^{z}}{\text{d}{\hat{\rho }}_{t}}\text{d}\mu (z)$ . The biggest technical problem is to ensure that ${\hat{\rho }}_{t}^{z}\ll {\hat{\rho }}_{t}$ , which is proven to be true if the collection of parametrized measures are μ a.e. mutually absolutely continuous. This holds for a class of conditional flows (induced by the conditional fields) of the form $\phi^{z}({\tilde{x}}_{0},t)=\sigma (t)x_{0}+m(t)z$ *,* where m,σ are selected appropriately ( σ(0)=m(T)=1,σ(1)=m(0)=0 ), which corresponds to conditional vector fields


(3.6) $$v^{z}(x,t)=\frac{\text{d}\log\sigma (t)}{\text{d}t}(x-m(t)z)+\frac{\text{d}m(t)}{\text{d}t}z.$$

In this practical case, the sequence of measures $\text{d}{\hat{\rho }}_{t}^{f}(g)$ admits known Gaussian closed form.

The relationship between the technique described in [29] and the other ones presented previously can be understood assuming that ${\hat{\rho }}_{t}=\rho_{T-t}$ , with A=−I/2 . Then ${\hat{\rho }}_{t}^{z}(x)=N_{\exp(-(T-t)/2)z,(1-\exp(-(T-t)))R}(x)$ . Notice that, in this comparison, the flow starts from a measure $\rho_{T}$ which is only *close* to a purely Gaussian measure. Then, we can explicitly identify the vector field v(x,t) from equation (2.13), after simple manipulation, as $v(x,t)=-Ax+\frac{1}{2}R▽\log\rho_{T-t}(x)$ , thanks to the content of the second form of the Fokker–Planck equation, as shown in equation (2.12).

This correspondence is useful in that the same conditions of theorem 2.2, which allow us to claim validity for the other class of models, are sufficient to ensure that the models considered in [29] are well defined. Indeed, other than integrability assumptions, the main assumption in Theorem 1 of [29] is that $\rho_{t}^{z}\ll \rho_{t}$ for μ a.e. z and almost every t . Under the same assumptions of theorem 2.2, this holds.

**Theorem 3.1.** *As in theorem 2.2, assume that* $\mu (R^{\frac{1}{2}}H)=1$ *and condition 2.2 holds. Then,* $\rho_{t}^{z}\ll \rho_{t}$ *for* μ *a.e.* z *and almost every* t .

*Proof*. We consider point (ii) of theorem 2.2, $\rho_{t}\ll \rho_{t}^{0}$ , which reads $\rho_{t}^{0}(B)=0\Rightarrow \rho_{t}(B)=0$ for any t∈(0,T] . Since $\rho_{t}(B)=\int \rho_{t}^{z}(B)\text{d}\mu (z)$ , we have $\rho_{t}(B)=0\Rightarrow \rho_{t}^{z}(B)=0$ , for μ a.e. z . Chaining the results, this implies that $\rho_{t}^{z}\ll \rho_{t}^{0}$ , for μ a.e. z . Since both measures are Gaussian, this implies their equivalence, $\rho_{t}^{z}∼\rho_{t}^{0}$ for μ− a.e. z . Then, for any $z,z^{\prime }$ *,* $\rho_{t}^{z}∼\rho_{t}^{z^{\prime }}$ , which is enough to prove that $\rho_{t}^{z}\ll \rho_{t}$ for μ a.e. z and almost every t , since (0,T] has full measure ([29], Theorem 2).∎

## 4. Parametric approximations

Our discussion so far has been rooted in the underlying assumption of having access to the key vector fields of the dynamics for the generation process: $▽\log\rho_{t}(x),▽\log\frac{\text{d}\rho_{i/N}}{\text{d}\nu_{i/N}}(x)$ and v(x,t) . Clearly, this is not the case in practice, for realistic (and unknown) μ . In all implementations, the true vector field is replaced by a *parametric* ( $\theta \in {\mathbb{R}}^{p}$ ) approximation $s_{\theta }(x,t)$ . Importantly, working in the functional domain calls for architectural choices which are suited for the infinite-dimensional domain. The most popular choice for *resolution invariant* architectures is the family of Neural (Fourier) Operators [67,68]. These architectures are extremely powerful in representational power but suffer from the drawback of requiring the input points to be collected on a regularly spaced grid [68], limiting their usage. Alternatively, transformers architectures [69] have been considered, by interpreting them as mappings between Hilbert spaces [70]. The flexibility of being capable of handling irregularly spaced grids comes at the cost that resolution invariance has to be *learned* during training, and it is not guaranteed by default for Neural Operators. Finally, it is worth mentioning the class of Implicit Neural Representations [71], which have the requested resolution-invariant properties, are known to be naturally good denoisers [72], but require meta-learning techniques for working properly [73]. Another possibility, not yet explored by the community, is to combine the different architectures mentioned, as done in the empirical work [74].

### (a) Loss functions and time discretization

While the selection of an appropriate architecture is an important endeavour, a fundamental aspect is the selection of appropriate loss functions for learning the parameters of such architectures. We then proceed by providing an overview of various loss functions employed in different approaches to infinite-dimensional diffusion models, highlighting their mathematical formulations and connections. Interestingly, all variants presented in the literature can be understood under the lens of learning to *denoise* a corrupted version of the input data.

The learning objective associated with equation (2.4) can be formulated as an evidence lower bound (ELBO) on the log-likelihood of the data (see [26] for full details). In particular, such ELBO arises from comparing the path measures of the forward and reverse diffusion processes, leveraging Girsanov’s theorem in infinite dimensions [37]. The discrepancy between the measure χ obtained with the approximated dynamics (and with initial conditions drawn from $\rho_{T}^{0}$ in place of $\rho_{T}$ ) and the true one can be expressed in Kullback–Leibler (KL) terms as


(4.1) $$\text{KL}(\mu ||\chi )\leq \frac{1}{2}E_{Q}\left[\int_{0}^{T}||R(s_{\theta }(X_{t},t)-▽\log\rho_{t}(X_{t}\;|\;X_{0}))|{|}_{R^{1/2}H}^{2}dt\right]+\text{KL}(\rho_{T}||\rho_{T}^{0})+\text{const}(\theta ),$$

where $\langle ▽\log\rho_{t}(x\;|\;x_{0})),e^{k}\rangle =\frac{\partial }{\partial x^{k}}\log\frac{\text{d}\rho_{t}(x^{k}\;|\;x_{0})}{\text{d}x^{k}}$ . In the linear case, the conditional score term has expression [26]


(4.2) $$R▽\log\rho_{t}(x\;|\;x_{0})=-RS(t{)}^{-1}\left(x-\exp(tA)x_{0}\right).$$

Minimizing the loss $C(\theta )=E_{Q}\left[\int_{0}^{T}||Rs_{\theta }(X_{t},t)+RS(t{)}^{-1}\left(X_{t}-\exp(tA)X_{0}\right)|{|}_{R^{\frac{1}{2}}H}^{2}dt\right]$ is then equivalent to minimizing the ELBO. Related losses, but with different preconditioning, are also considered in [24,31,45]. A typical parametrization chosen for $s_{\theta }(x,t)$ is in the form $S(t{)}^{-1}\left(X_{t}-\exp(tA)n_{\theta }(x,t)\right)$ , from which


$$C(\theta )=E_{Q}\left[\int_{0}^{T}||RS^{-1}(t)\exp(tA)\left(s_{\theta }(X_{t},t)-X_{0}\right)|{|}_{R^{\frac{1}{2}}H}^{2}dt\right]$$

and is thus evident that the optimal solution for the parametric network $n_{\theta }(x,t)$ is the ‘denoiser’ $E_{\mathbb{Q}}[X_{0}\;|\;X_{t}=x]$ .

While the continuous-time framework provides a strong theoretical foundation for understanding diffusion processes, practical implementations often rely only on discrete-time approximations. In this setting [28], the first work in chronological order in considering Hilbert space valued generative diffusion models, the continuous path of measures $(\rho_{t}{)}_{t\in [0,T]}$ is replaced by the finite sequence of measures, and the forward and reverse diffusion processes are approximated using discrete-time transitions, from which an analogous ELBO to equation (4.1) is obtained (where effectively, integration over t is substituted with a finite sum over the discrete steps of the generation chain). One important aspect of this approach is that it automatically includes in the lower bound the effect of discretization of the SDEs, which is instead not included explicitly in the continuous time methods (when implemented, such methods clearly need to include some form of numerical integration [75–81]). Whenever the number of numerical integration steps is limited, in the finite-dimensional literature of diffusion models, it has been observed that optimizing directly the discrete-time lower bound provides better results [82]. While the same should hold in principle also in the infinite-dimensional settings, to the best of our knowledge, none of the work present in the literature has directly explored the problem.

Other families of approaches [29,30] adopt similar techniques, where a conditional version of the target field is considered for learning the vector fields. In particular, for learning $▽\log\frac{\text{d}\rho_{i/N}}{\text{d}\nu_{i/N}}(x)$ through the equality formalized by equation (2.8), the following loss is considered: $C(\theta )=\sum_{i=1}^{N}E_{Q}\left[{\left\|\exp(iT/NA)X_{0}-s_{\theta }(X_{iT/N},iT/N)\right\|}^{2}\right]$ . Each term in the loss corresponds to the mismatch between the true Langevin drift and the approximated one. Interestingly, in principle, the scheme in [30] could still achieve very good performance while having bad approximations for all the terms but the last one, provided the last chain is run for long enough. A quantitative analysis of such discrepancy is an interesting avenue for future work, but it is worth mentioning that the study of the convergence rate of Langevin dynamics in infinite dimensions is much more challenging than for the finite-dimensional case [19]. Finally, in [29], the considered loss is $C(\theta )=E_{Q}\left[\int_{0}^{T}||s_{\theta }(X_{t},T-t)-v^{X_{0}}(X_{t},T-t){||}^{2}\right]$ which, upon noticing that $v^{X_{0}}(X_{t},T-t)=\frac{\text{d}\log\sigma (t)}{\text{d}t}(X_{t}-m(t)X_{0})+\frac{\text{d}m(t)}{\text{d}t}X_{0}$ (equation (3.6)), can again be interpreted as a denoising objective.

## 5. Applications and extensions

One of the primary applications of diffusion models in function spaces is data generation at arbitrary resolutions. This capability is essential in domains where high-resolution or continuous data representations are required. For example, in the fields of image and audio synthesis, where traditional generative models operate in discrete, finite-dimensional spaces, diffusion models in function spaces can inherently capture and generate data that varies continuously across scales. Recent works have demonstrated that these models can be particularly powerful for generating high-resolution images or audio waveforms, with Neural Operators, INRs or Transformer architectures providing a way to model functional data while maintaining resolution invariance [26,27,31,50,83]. Other authors have mainly focused on time series modelling under the functional formalism [28,29], or focused on new methodologies for physical sciences simulations and inverse problems [30]. In general, function space diffusion models can be applied to any modality on which finite-dimensional techniques have been applied, and beyond. We comment that, other than the obvious application scenario of generative modelling, variants of these diffusion models have also been applied for other problems, which we overview in the following.

### (a) Diffusion bridges and optimal control

For example, the works presented in [32,33,50] introduce an approach for simulating nonlinear diffusion bridges in infinite-dimensional spaces. The method involves considering a diffusion process (with measure ℚ )


(5.1) $$\text{d}X_{t}=(AX_{t}+f(X_{t}))\text{d}t+\text{d}W_{t},X_{0}=x_{0},$$

and proving, through a generalization of Doob’s h-transform [84] to infinite dimensions, that the representation of an SDE corresponding to a process with path measure ${\mathbb{Q}}^{\chi }$ , where $Q^{\chi }(\cdot )=\int Q(\cdot \;|\;X_{T}=y)\text{d}\chi (y)$ , $\chi \ll \rho_{T}$ , is again an SDE in the form


(5.2) $$\text{d}X_{t}=(AX_{t}+f(X_{t})+R▽\log h(X_{t},t))\text{d}t+\text{d}W_{t},X_{0}=x_{0},$$

where $h(x,t)=E_{Y∼\chi }E_{Q}[1_{\text{d}Y}(X_{T})\;|\;X_{t}=x]=\int \text{d}\chi (z)\text{d}Q(X_{T}=z\;|\;X_{t}=x)$ . Unfortunately, excluding simple cases, knowledge of ▽log⁡h(x,t) is out of reach. Even worse, *a priori*, it is not even known whether h(x,t) is differentiable and the problem is consequently well defined.

In the linear case, under the assumption $\exp(tA)z\in S_{t}^{\frac{1}{2}}H$ , the derivative exists and furthermore $\rho_{t}^{z}\ll \rho_{\infty }$ , with known differentiable density $q_{t}(z,x)$ (for the exact form and a proof refer to [59,85,86]).^1^ Then, noticing the time homogeneity of the problem $h(x,t)=\int \text{d}\chi (z)\text{d}Q(X_{T-t}=z\;|\;X_{0}=x)$ $\;=\int \text{d}\chi (z)\text{d}\rho_{T-t}^{x}(z)$ $\;=\int \text{d}\chi (z)q_{T-t}(x,z)\text{d}\rho_{\infty }(z)$ , that is a form allowing for the computation of ▽log⁡h(x,t) , which coincides with $▽\log\rho_{T-t}(x)$ [50]. In [50], such formalism is investigated in deeper detail and adopted for building bridges between broader modifications of ℚ , for example for the measure $\int \text{d}\chi (y)\text{d}\zeta (x)Q(\cdot \;|\;X_{0}=x,X_{T}=y)$ . These extensions allow us, for example, to perform generic bridge matching and implement Bayesian learning in function space [50]. Other than for generative modelling purposes or stochastic control, stochastic bridges are also useful in other contexts, such as phylogenetic shape analysis, which models the stochastic change in animals' morphometry over time [87].

When considering nonlinear SDEs, the situation becomes more complex as, in general, it is harder to prove the existence of the derivative of h(x,t) *,* and it is impossible to sample from $\mathbb{Q}(X_{T}=\cdot \;|\;X_{t}=x)$ without having first constructed the bridge, which introduces a circular dependency. The authors in [32,33] adopt the strategy of time reversal to solve such problems and simplify the implementation.

For the following passages to be valid, it is important to *assume* the existence of the various Radon–Nikodym derivatives involved, whose proof is in general a far from trivial task. We consider for simplicity of exposition deterministic terminal conditions, i.e. ${\mathbb{Q}}^{\chi }(\cdot )=\mathbb{Q}(\cdot \;|\;X_{T}=y)$ , and consequently $h(x,t)=\text{d}Q(X_{t}=x\;|\;X_{T}=y)$ . By writing $\text{d}Q(X_{t}=x\;|\;X_{T}=y)=\frac{\text{d}Q(X_{t}=x\;|\;X_{T}=y)}{\text{d}Q(X_{t}=x)}\text{d}Q(X_{T}=y)$ , we can explicitly write $▽\log h(x,t)=▽\log\frac{\text{d}Q(X_{t}=x\;|\;X_{T}=y)}{\text{d}Q(X_{t}=x)}$ , and in particular then $\langle R▽\log h(x,t),e^{k}\rangle =r^{k}\frac{\partial }{\partial x^{k}}\left(\log\left(\frac{\text{d}Q(X_{t}^{k}=x^{k}\;|\;X_{T}=y,X_{t}^{j\neq k}=x^{j\neq k})}{\text{d}Q(X_{t}^{k}=x^{k}\;|\;X_{t}^{j\neq k}=x^{j\neq k})}\right)\right)$ . The time reversal of equation (5.2) involves the distribution $\rho_{t}^{\chi }$ associated to ${\mathbb{Q}}^{\chi }$ , and consequently $\langle R▽\log\rho_{t}^{\chi }(x),e^{k}\rangle =r^{k}\frac{\partial }{\partial x^{k}}\left(\log\left(\frac{\text{d}\rho_{t}(X_{t}^{k}=x^{k}|X_{t}^{j\neq k}=x^{j\neq k},X_{T}=y)}{\text{d}x^{k}}\right)\right)$ . Then $-R▽\log h(x,t)+R▽\log\rho_{t}^{\chi }(x)=R▽\log\rho_{t}(x)$ . Conceptually, this implies the following: to simulate a bridge in the form of equation (5.2), it is possible to learn $R▽\log\rho_{t}(x)$ from equation (5.1), which has no constraints on the ending value and is thus an easier task, and then simulate the backward dynamics of equation (5.2), where initial conditions are known and the drift term is equal to $-Ax-f(x,T-t)+R▽\log\rho_{t}(x)$ . For full details and formal validity of the derivations, we refer the reader to [32,33].

### (b) Bayesian inverse problems

Finite-dimensional diffusion models have been explored as strong priors for Bayesian inverse problems in multiple domains [51,52]. The extension of these techniques to the Hilbert space settings has been explored by the authors in [24,25], in the case of linear and nonlinear observations, respectively. Considering the linear observation problem, it is assumed to have access to $Y=P(X_{0})+B$ , where $P:H\to R^{M}$ is a *linear* operator and B is an additive noise random variable with a density ψ w.r.t. the Lebesgue measure. Then, if μ≪N(0,R) (note that the work in [24] considers a more generic $\tilde{R}$ ) it is possible to show that the following scheme


(5.3) $$\text{d}{\hat{X}}_{t}=\frac{1}{2}{\hat{X}}_{t}-(1-\exp(-t){)}^{-1}\left(x-\exp\left(-\frac{t}{2}\right)E\left[X_{0}\;|\;X_{T-t}={\hat{X}}_{t},Y\right]\right)+\text{d}{\hat{W}}_{t},{\hat{X}}_{0}∼Q(X_{T}\;|\;Y)$$

with ${\hat{W}}_{t}$ being a R -Wiener process provides, at time t=T , a valid sample from the posterior distribution $\mathbb{Q}(X_{0}\;|\;Y)$ . The result is obtained considering the particular case of equation (2.1) where f=0 and $A=-\frac{1}{2}I$ . The proof requires mimicking the result of [45], extending it for the conditional case. The authors extend their work in two directions: first, considering the case of nonlinear operator P , and second, performing conditional sampling with only knowledge of the unconditional $E\left[X_{0}\;|\;X_{t}\right]$ (thus allowing the technique to adopt any pre-trained unconditional model as in [88]). A simplified expression of the scheme proposed in [25] reads


(5.4) $$\text{d}{\hat{X}}_{t}=\frac{R}{2}▽\log\rho_{\tau }({\hat{X}}_{t})+\frac{R}{2}▽\log(\psi (Y-P({\hat{X}}_{t})))+\text{d}{\hat{W}}_{t},{\hat{X}}_{0}∼Q(X_{T}\;|\;Y),$$

where τ>0 is a parameter which is annealed from large to small values. Given the similarity between equations (5.4) and (3.3), it is natural to interpret the former as an infinite-dimensional generalization of the conditioning trick [89], which in finite dimension reads $▽\rho_{\tau }(x\;|\;y)=▽\rho_{\tau }(x)+▽Q(y\;|\;x)$ . We invite the reader to refer to [25] for generalizations of this scheme and quantitative bounds on the quality of approximated posterior.

## 6. Conclusions

In this survey, we have explored the extension of diffusion-based generative models from finite-dimensional settings to infinite-dimensional function spaces, focusing on the theoretical underpinnings and practical implementations of such models in Hilbert spaces. By leveraging SDEs and the properties of time reversal in infinite dimensions, we have highlighted how these models can effectively generate function-valued data, offering promising applications in domains that require high-resolution generative capabilities.

While significant progress has been made in adapting diffusion models to infinite-dimensional settings, several challenges remain open. One of the key difficulties is ensuring the efficient computation of generative dynamics in function spaces without sacrificing resolution invariance. Approximations and parametric methods, such as those based on neural Fourier operators and transformer architectures, have shown promise, but further improvements are necessary to fully realize the potential of these models in practical settings.

An interesting direction for future research would be the exploration of perturbation-based scenarios for infinite-dimensional diffusion models, akin to those already developed for finite-dimensional cases. For instance, works such as [90–93] have successfully extended finite-dimensional diffusion models to account for different perturbative settings. Applying these methods to function spaces could open new avenues for developing more robust generative models that operate under complex data perturbations or in noisier, real-world applications. This extension could provide stronger priors for solving inverse problems, denoising functional data or addressing generative tasks in physical sciences. Alternatively, it would be interesting to draw the functional domain equivalent of the alternatives to diffusion in finite dimensions [47,94,95].

Moreover, the theoretical understanding of how these perturbation techniques interact with the infinite-dimensional structure remains an open problem. As with finite-dimensional models, establishing precise conditions for the stability, convergence and efficiency of these models in infinite dimensions will be critical for both theoretical development and their practical utility.

In conclusion, infinite-dimensional diffusion models represent a powerful and flexible approach to generative modelling in functional domains. Their potential in practical applications is clear but under-explored, and further extensions, especially along the lines of perturbation methods, could significantly advance both the theory and applications of functional models.

## Data Availability

This article has no additional data.

## Footnotes

^1^
Notice that this condition is in general not compatible with the ones guaranteeing the existence of a time reversal as defined by [57].

## Appendix A. Detailed conditions for theorem 2.1

We provide a complete list of assumptions from [65], where the authors consider a generalization of the infinite system of equations

(A 1)
$$\text{d}Y_{t}^{k}=b^{k}(Y_{t},t)\text{d}t+\sigma^{k}(t)\text{d}W_{t}^{k},\;k\in N,$$

where $W_{t}^{k}$ are Brownian motions, with $E_{\mathbb{Q}}[W_{t}^{k}W_{s}^{j}]=\delta (k-j)r^{k}\min(s,t)$ *,* δ in the Kronecker sense. For clarity, we denote the law of the process by $\text{d}\rho_{t}^{Y}(y)$ when necessary. The system studied in [65] evolves in the space of weighted square-summable sequences,

$$l_{2}^{r}(\mathbb{N})\overset{\text{ def}}{=}\{y\in {\mathbb{R}}^{\mathbb{N}}:\sum r^{i}(y^{i}{)}^{2}<\infty \},$$

while the original process X is defined in $l_{2}(\mathbb{N})$ . Since these two spaces are isomorphic, results from one setting can be translated into the other via simple algebraic manipulations. We now list the necessary assumptions for ensuring the existence of the reverse process. We state the assumptions in a simplified form, which leverages the fact that in this work we consider σ to be independent from y .

**Assumption A.1.** (*Assumption H1 in* [65]). There exists a finite positive constant K such that $\forall y,z\in l_{2}^{r}(\mathbb{N}):$

$$\begin{array}{rl} & \underset{t}{\sup}\left\{\sum_{i}r^{i}{\left(b^{i}(y,t)\right)}^{2}+\sum_{i}{\left(r^{i}\right)}^{2}{\left(\sigma^{i}(t)\right)}^{2}\right\}\leq K\left(1+\sum_{i}r^{i}{\left(y^{i}\right)}^{2}\right)\\ & \underset{t}{\sup}\left\{\sum_{i}r^{i}{\left(b^{i}(y,t)-b^{i}(z,t)\right)}^{2}\right\}\leq K\sum_{i}r^{i}{\left(y^{i}-z^{i}\right)}^{2}. \end{array}$$

**Assumption A.2.** (*Assumption H2 of* [65]). For each k∈ℕ and t∈[0,T] , the coefficients $b^{k}(y,t)$ depend on y on at most a number of finite coordinates.

**Assumption A.3.** (*Assumption H4 of* [65]). There exists a positive constant K such that $\forall y,z\in l_{2}^{r}(\mathbb{N})$

$$\underset{t}{\sup}\left\{\sum_{i}{\left(r^{i}\right)}^{2}{\left(b^{i}(y,t)-b^{i}(z,t)\right)}^{2}\right\}\leq K\sum_{i}{\left(r^{i}\right)}^{2}{\left(y^{i}-z^{i}\right)}^{2}.$$

**Assumption A.4.** (*Assumption H5 of* [65]). There exists an increasing sequence of finite subsets I(i),i≥0 which cover ℕ (i.e. $\cup_{i}I(i)=\mathbb{N}$ ) such that ∀i∈ℕ,C>0 , there exists a constant K(i,C) for which

$$\underset{t}{\sup}\underset{k\in I(i)}{\sup}\sup\{|b^{k}(y,t)|+\sum_{j\in N}r^{k}|\sigma^{j,k}(t)|:\underset{m\in I(i)}{\sup}|y^{m}|\leq C\}\leq K(i,C).$$

**Assumption A.5.** (*parts (iii,iv) of Theorem 4.3. in* [65]). Suppose that the initial condition $Y_{0}\in l_{2}^{r}(\mathbb{N})$ .

— *Assume that the conditional law of* $y^{i}$ *given* $y^{j},j\neq i$ *has density with respect to Lebesgue measure on* ℝ *, i.e. the conditional densities* $\frac{\text{d}\rho_{t}^{Y}(y^{i}\;|\;y^{j\neq i})}{\text{d}y^{i}}$ *exist*.
— *Fix a finite subset* J⊂ℕ . *For every positive* $t_{0}$ *, every* $D\in \{(\prod_{j\in J}K_{j})\times (\prod_{j\notin J}R),K_{j}\text{ compact in }R\}\cap l_{2}^{r}(N)$ *, and every* i∈ℕ *assume that*
  $$\int_{t_{0}}^{1}\int_{D}|r^{i}(\sigma^{i}(t){)}^{2}\frac{\partial }{\partial y^{i}}(\frac{\text{d}\rho_{t}^{Y}(y^{i}\;|\;y^{j\neq i})}{\text{d}y^{i}})|\text{d}y^{i}\rho_{t}^{Y}(\text{d}y^{j\neq i})\text{d}t<\infty .$$

Given the assumptions outlined above, we can now formally state the main result of Theorem 4.3 in [65]:

**Theorem A.1.** *Consider the system in* equation (A 1). *Suppose assumptions* A.1*,* A.2*,* A.3*,* A.4*, and* A.5 *hold. Then the time reversal of* equation (A 1) *is a solution of the* SDE *taking values in* $l_{2}^{r}(\mathbb{N})$

(A 2)
$$\text{d}{\hat{Y}}_{t}^{k}=-b^{k}({\hat{Y}}_{t},T-t)+r^{k}(\sigma^{k}(T-t){)}^{2}\frac{\partial }{\partial y^{k}}\log\left(\frac{\text{d}\rho_{T-t}^{Y}({\hat{Y}}_{t}^{k}|{\hat{Y}}_{t}^{j\neq k})}{\text{d}y^{k}}\right)\text{d}t+\sigma^{k}(T-t)\text{d}{\hat{W}}_{t}^{k}.$$

As a direct corollary, we consider the system of equations in $l_{2}(\mathbb{N})$ ,

$$\text{d}X_{t}^{k}=a^{k}(X_{t})+f^{k}(X_{t},t)\text{d}t+\text{d}W_{t}^{k},\;k\in N,$$

which admits strong solutions. We introduce the coordinate transformation $y^{k}=(r^{k}{)}^{-\frac{1}{2}}x^{k}$ , yielding the equivalent system

(A 3)
$$\text{d}Y_{t}^{k}=\underset{b^{k}(Y_{t},t)}{\underbrace{(r^{k}{)}^{-\frac{1}{2}}\left(a^{k}(R^{\frac{1}{2}}Y_{t})+f^{k}(R^{\frac{1}{2}}Y_{t},t)\right)}}\text{d}t+\underset{\sigma^{k}(t)}{\underbrace{(r^{k}{)}^{-\frac{1}{2}}}}\text{d}W_{t}^{k},\;k\in N,$$

which evolves in $l_{2}^{r}(\mathbb{N})$ since

$$\sum_{i}(x^{i}{)}^{2}=\sum_{i}r^{i}{\left((r^{i}{)}^{-\frac{1}{2}}x^{i}\right)}^{2}=\sum_{i}r^{i}(y^{i}{)}^{2}.$$

For this transformed system, we verify that assumptions A.1, A.2, A.3, A.4 and A.5 hold. If these conditions are satisfied, the time-reversed process of equation (A 3) satisfies equation (A 2). Since the transformation satisfies $\text{d}{\hat{X}}_{t}^{k}=(r^{k}{)}^{\frac{1}{2}}\text{d}{\hat{Y}}_{t}^{k}$ , the reversed system takes the form

(A 4)
$$\text{d}{\hat{X}}_{t}^{k}=-\left(a^{k}({\hat{X}}_{t})+f^{k}({\hat{X}}_{t},T-t)\right)+(r^{k}{)}^{\frac{1}{2}}\left(\frac{\partial }{\partial y^{k}}\log\left(\frac{\text{d}\rho_{T-t}^{Y}(y^{k}|y^{j\neq k})}{\text{d}y^{k}}\right)\right){|}_{y=R^{-\frac{1}{2}}{\hat{X}}_{t}}\text{d}t+\text{d}{\hat{W}}_{t}^{k}.$$

Applying the change of variable formula for measures, we obtain $\frac{\text{d}\rho_{t}^{Y}(y^{k}|y^{j\neq k})}{\text{d}y^{k}}=(r^{k}{)}^{\frac{1}{2}}\frac{\text{d}\rho_{t}^{X}(x^{k}|x^{j\neq k})}{\text{d}x^{k}}$ , which leads directly to the form given in equation 2.4.

### A.1. The linear case

We now show that condition 2.3 ensures the validity of assumptions A.1, A.2, A.3 and A.4. Under this condition, the forward system satisfies

(A 5)
$$\text{d}X_{t}^{k}=a^{k}X_{t}^{k}\text{d}t+\text{d}W_{t}^{k},\;k\in N,$$

which admits a strong solution in $l_{2}(\mathbb{N})$ (see condition 2.1). Transforming via $y^{i}=(r^{i}{)}^{-\frac{1}{2}}x^{i}$ , we obtain

(A 6)
$$\text{d}Y_{t}^{k}=a^{k}Y_{t}^{k}\text{d}t+(r^{k}{)}^{-\frac{1}{2}}\text{d}W_{t}^{k},\;k\in N.$$

Under this transformation, assumption A.1 reduces to

$$\begin{array}{rl} & \forall y\in l_{2}^{r}(N),\;\sum_{i}r^{i}{\left(a^{i}\right)}^{2}{\left(y^{i}\right)}^{2}+\sum_{i}r^{i}\leq K\left(1+\sum_{i}r^{i}{\left(y^{i}\right)}^{2}\right),\\ & \forall y,z\in l_{2}^{r}(N),\;\sum_{i}r^{i}{\left(a^{i}\right)}^{2}{\left(y^{i}-z^{i}\right)}^{2}\leq K\sum_{i}r^{i}{\left(y^{i}-z^{i}\right)}^{2}. \end{array}$$

Since b and σ are independent of t , these assumptions are satisfied, given that $\sum_{i}r^{i}=K_{1}$ and

$$\sum_{i}r^{i}(a^{i}{)}^{2}(y^{i}{)}^{2}\leq M^{2}\sum_{i}r^{i}(y^{i}{)}^{2},\;\sum_{i}r^{i}(a^{i}{)}^{2}(y^{i}-z^{i}{)}^{2}\leq M^{2}\sum_{i}r^{i}(y^{i}-z^{i}{)}^{2},$$

where M is the constant from condition 2.3. Assumption A.3 is verified similarly, while A.2 and A.4 follow from the diagonality of A . In particular for A.4, consider I(i)={i} , from which the assumption is satisfied if $\sup\{|a^{i}y^{i}|+(r^{i}{)}^{\frac{1}{2}}:|y^{i}|\leq C\}\leq K(i,C),\forall i\in \mathbb{N}$ *,* which clearly holds since $a^{i}$ is bounded.

Using again the relation $\frac{\text{d}\rho_{t}^{Y}(y^{k}|y^{j\neq k})}{\text{d}y^{k}}=(r^{k}{)}^{\frac{1}{2}}\frac{\text{d}\rho_{t}^{X}(x^{k}|x^{j\neq k})}{\text{d}x^{k}}$ , along with the results in theorem 2.2(iii), we establish that the integrability condition in assumption A.5 holds, since the time interval considered is (0,1] .
